# Patients' views on primary care multidisciplinary teams in Scotland: a mixed-methods evaluation

**DOI:** 10.3399/BJGPO.2023.0200

**Published:** 2024-09-18

**Authors:** Kieran D Sweeney, Eddie Donaghy, David Henderson, Harry HX Wang, Andrew Thompson, Bruce Guthrie, Stewart W Mercer

**Affiliations:** 1 Usher Institute, College of Medicine and Veterinary Medicine, University of Edinburgh, Edinburgh, United Kingdom; 2 School of Public Health, Sun Yat-Sen University, Guangzhou, China; 3 School of Social and Political Science, University of Edinburgh, Edinburgh, United Kingdom

**Keywords:** multidisciplinary teams, primary care reform, healthcare inequalities, healthcare disparities, primary health care, Scotland

## Abstract

**Background:**

Expanding primary care multidisciplinary teams (MDTs) was a key component of the 2018 Scottish GP contract, with more than 4700 MDT staff appointed since then.

**Aim:**

To explore patients’ views on primary care MDT expansion in Scotland.

**Design & setting:**

A mixed-methods evaluation, which included a postal survey and semi-structured telephone interviews with patients in Scotland.

**Method:**

A survey was undertaken of patients who had recently consulted a GP in deprived urban, affluent urban, and remote and rural areas, assessing awareness of five MDT roles and attitudes towards receptionist signposting. In addition, 30 individual interviews were conducted, exploring patients' MDT-care experiences.

**Results:**

Of 1053 survey responders, most were unaware of the option of MDT rather than GP consultations for three out of five roles (69% unaware of link worker appointments; 69% mental health nurse; and 58% pharmacist). Reception signposting was less popular in deprived urban areas (34% unhappy versus 29% in remote and rural versus 21% affluent urban; *P*<0.001), and in patients with multimorbidity (31% unhappy versus 24% in non-multimorbid; *P*<0.05). Just over two-thirds of interviewees had multimorbidity and almost all reported positive MDT-care experiences. However, MDT care was generally seen as a supplement rather than a substitute for GP care. Around half of patients expressed concerns about reception signposting. These patients were more likely to also express concerns about GP access in general. Both of these concerns were more common in deprived urban areas than in remote and rural or affluent urban areas.

**Conclusion:**

MDT care has expanded in Scotland with limited patient awareness. Although patients understand its potential value, many are unhappy with reception signposting to first-contact MDT care, especially those in deprived urban areas living with multimorbidity. This represents a barrier to the aims of the new GP contract.

## How this fits in

The expansion of primary care MDTs is a key component of the new GP contract in Scotland and a common feature of primary care reforms globally. By increasing the amount of care provided by healthcare professionals other than GPs, the new Scottish contract aimed to improve the quality and accessibility of care, while freeing-up time for GPs to spend with the most complex patients, thereby also addressing health inequalities. This mixed-methods study found limited patient awareness of MDT expansion, and, while patients recognise its potential value, accessible GP care remains a priority, and MDT care is viewed as a supplement rather than a substitute for this. Reception signposting to MDT care is particularly unpopular with patients with multimorbidity in deprived areas. These findings represent a barrier to the aims of the new GP contract, which must be addressed if continued MDT expansion is to benefit those with the most complex needs.

## Introduction

Multidisciplinary teams (MDTs) are an increasingly common feature of primary care systems worldwide.^1–5^ Services that bring together a range of healthcare professionals to deliver care alongside GPs are assumed to be better equipped to provide accessible and comprehensive primary care,^4–8^ especially given the global shortage of GPs.^9,10^ MDT-based models of care (also referred to as multiprofessional teams, interdisciplinary care, interprofessional care, and interprofessional collaborative practice) have been shown in some studies to improve health outcomes and reduce healthcare costs.^11,12^ Accordingly, the expansion of MDTs has been a common feature of recent primary care reforms in high-income countries, as health systems seek to address challenges of increasing multimorbidity, population ageing, rising costs, and growing health inequalities.^13^

In Scotland, the expansion of primary care MDTs was a key part of the new GP contract introduced in April 2018 against a background of service redesign.^14^ In April 2016, the Quality Outcome Framework pay-for-performance system was abolished and GP practices began to be organised into geographic clusters, promoting shared quality improvement. The 2018 GP contract formalised these changes and further refocused the role of the GP as the expert medical generalist leading a MDT of professionals, including practice nurses, advanced nurse practitioners, physiotherapists, mental health nurses, pharmacists, and community link workers.^14^ By increasing the amount of care provided by professionals other than GPs, the contract aimed to improve the quality and accessibility of care for patients, while reducing GP workload.^14^ In turn, this was intended to free-up GP time to spend with patients with complex needs. Between 2018 and 2023, 4730 whole-time equivalent new MDT staff were appointed in primary care in Scotland, covering 911 practices.^15,16^

As well as addressing pressures of increasing multimorbidity and population ageing, a stated aim of the 2018 GP contract was addressing health inequalities. These are widening in Scotland, with disparities not only across socioeconomic groups, but also by sex, ethnicity, and geography.^17^ The latter is of particular relevance in Scotland, where more than 15% of the population live in remote and rural areas.^17,18^ The expansion of primary care MDTs (including community link workers who focus on connecting patients to non-medical community resources in deprived areas) was central to this aim of addressing health inequalities. The extent to which this has been achieved is unknown, but our recent research suggests it has not been attained, with GPs in deprived areas reporting that the new contract has failed to free-up GP time to spend with complex patients, and those in rural areas viewing it as too ‘city centric’.^19–21^

Most evaluations of MDT-based models of primary care have focused on health outcomes and costs.^11,12^ Our previously published qualitative evaluations have explored the views of a range of professionals with regard to the new GP contract in Scotland,^19,20^ but the views and experiences of patients remains under-explored.^8,13,22,23^ This study aims to assess the awareness, attitudes, and experiences of patients in Scotland regarding primary care MDT expansion, and, given the aims of the new GP contract with respect to inequalities, to look for differences between socio-geographic groups.

## Method

### Study design

The overall study design was mixed methods, with a postal survey of patients who had recently consulted a GP, followed by individual telephone interviews with a sub-sample of responders.

### Sampling and recruitment

Three regional health boards (out of 14 across Scotland) were selected to give a range of geographic and socioeconomic characteristics. Four clusters were recruited from each of these health boards, and one practice recruited from each cluster. Practices were grouped according to whether they served mainly deprived urban (*n* = 6), affluent urban (*n* = 2) or remote or rural (*n* = 4) areas. A random sample of 6291 adult patients who had consulted a GP within the past 30 days were identified from practice records.

Surveys were sent with a cover letter, which included an optional expression-of-interest form to identify potential participants for follow-up qualitative interviews. Surveys were posted between 31 August and 15 September 2022. Collection of responses ran until 30 November 2022. Interviews were conducted between 26 October 2022 and 11 January 2023.

A total of 1053 questionnaire responses were received (overall response rate 17%). Response rates were higher in affluent urban practices (27%) than in remote and rural areas (20%) and deprived urban areas (12%). Of those who returned expression-of-interest forms, 30 patients were purposively sampled for interview (10 from each health board), with two-thirds aged ≥65 years and two-thirds with multimorbidity (two or more chronic conditions).

### Data collection

The questionnaire collected sociodemographic information, including responders’ age, sex, and postcode, which was used to define individual Scottish Index of Multiple Deprivation (SIMD) decile, with one being the most deprived 10% and 10 the least deprived 10%.^24^ Multimorbidity was assessed using a checklist of 17 common chronic conditions, with space to add additional conditions not listed, as in our previous studies.^25,26^

The questionnaire included several validated and bespoke items assessing health characteristics, patterns of consulting and consultation experience, results from which have been published separately.^21^ For the present paper, the questionnaire included bespoke items (Table 1) assessing responders’ awareness of the possibility of being offered an appointment with the following five MDT professionals: a nurse (including practice nurse, advanced nurse practitioner, or the support role of healthcare assistant); a mental health nurse based at the practice; a physiotherapist; a pharmacist or pharmacy technician based at the practice; and a community link worker or welfare adviser. Responders were also asked how happy they would feel (using a four-point Likert scale) if they were asked about their health concern by the receptionist for the purpose of signposting, if appropriate, to an MDT professional without seeing a GP first.

**Table 1.** Questions from survey covering MDT care and reception signposting

1. Due to recent changes in how general practice is organised in Scotland, are you aware that when you call your general practice you could (depending on your health problem) be offered an appointment with one of the following, instead of with a GP?			
a. A Nurse (eg, general practice nurse, an advanced nurse practitioner, or health care assistant)			
I am aware of this □	I don’t know about this □		
b. A Mental Health nurse based in the practice			
I am aware of this □	I don’t know about this □		
c. A Physiotherapist			
I am aware of this □	I don’t know about this □		
d. A pharmacist or pharmacy technician based in the practice			
I am aware of this □	I don’t know about this □		
e. Someone who provides advice or links to other services (eg, a community link worker, a welfare advisor)			
I am aware of this □	I don’t know about this □		
2. When you phone your practice about a health concern, how happy or unhappy would you be for the receptionist to ask you questions and to signpost you to the person (from the list above) that is most appropriate for your needs without seeing the GP first? (Please select one answer)			
Very happy □	Quite happy □	Quite unhappy □	Very unhappy □

One-to-one, semi-structured telephone interviews lasting 40–60 minutes were conducted with the 30 selected patients. Interviews were audio-recorded and transcribed verbatim. The interview topic guide covered patients’ views and experiences regarding access to appointments, GP care, MDT care, telephone consultations, and the impact of the COVID-19 pandemic (Supplementary Box S1). The present paper focuses on MDT care and reception signposting, with other results published separately.^27^

### Data analysis

Descriptive analysis of the questionnaire results was performed using SPSS (version 27). Differences between the three population groups (deprived urban, affluent urban, remote and rural) were assessed using the appropriate parametric or non-parametric tests (analysis of variance [ANOVA] and Kruskal–Wallis, respectively), depending on the distribution of the variables, with further pairwise comparisons conducted (using independent t-tests or Mann–Whitney tests) where a significant difference was found on three-way testing. Differences were also assessed according to age and multimorbidity.

Thematic analysis was conducted on the interview transcripts to identify common themes in patients’ views and experiences of MDT care and reception signposting, and to identify similarities and differences according to age, multimorbidity status, and population group. Three authors (ED, SWM, KS) independently identified codes based on individual analysis of selected interview transcripts, arriving at a common coding framework through discussion. Transcripts were coded using NVivo (version 12) by ED and KS. Six phases of thematic analysis (as outlined by Braun and Clarke)^28^ were applied by ED and KS as follows: familiarisation with the data; generation of initial codes; searching for themes; reviewing themes; defining and naming themes; and summarising themes for a final report.

Integration and synthesis of findings from the quantitative and qualitative components of this study were conducted by KS and SWM, according to Farmer and colleagues’ triangulation protocol, using a convergence coding matrix to identify areas of convergence, divergence, and silence.^29,30^

## Results

### Survey results

The characteristics of survey responders, and comparison with non-responders, are described in our previous paper and summarised in Supplementary Table S1.^21^ Survey results are summarised in Tables 2 and 3. Overall, a minority of responders were aware of link workers (31%), mental health nurses (31%), or pharmacists (42%). Awareness was slightly higher for physiotherapists (58%) and much higher for nurses (86%). The affluent urban group reported lower awareness of all MDT roles than both the deprived urban and remote and rural groups (*P*<0.001), except for nursing staff, where deprived urban and affluent urban groups were similar. Awareness of nursing, physiotherapy, and pharmacy staff was significantly higher in remote and rural areas than in both other groups (*P*-values <0.001,<0.001, and 0.003, respectively), while awareness of link workers and mental health nurses were similar between deprived urban and remote and rural areas. Awareness of physiotherapy and pharmacy roles was also higher in patients with multimorbidity than those without (*P*-values <0.001 and 0.017 respectively). Patients aged ≥65 years were significantly more likely to be aware of the nurse role and less likely to be aware of the link worker role than those aged <65 years (*P-*values 0.016 and 0.007 respectively).

**Table 2.** Awareness of MDT roles in participating patients from affluent urban, deprived urban, and remote and rural areas

	Remote and rural (RR) N = 332 % (*n*)	Affluent urban (AU) N = 273 % (*n*)	Deprived urban (DU) N = 448 % (*n*)	Overall 3-way comparison (*P*-value)	2-way group comparisons (*P*-value)	
Aware of option of seeing the following MDT roles:						
Nurse (or ANP or HCA)	94.4 (306)	81.8 (220)	82.6 (356)	<0.001	DU versus AU	0.784
					AU versus RR	<0.001
					DU versus RR	<0.001
Mental health nurse	38.2 (116)	21.0 (55)	33.2 (140)	<0.001	DU versus AU	<0.001
					AU versus RR	<0.001
					DU versus RR	0.166
Physiotherapist	72.3 (225)	41.0 (109)	58.7 (247)	<0.001	DU versus AU	<0.001
					AU versus RR	<0.001
					DU versus RR	<0.001
Pharmacist (or technician)	52.4 (162)	32.2 (86)	41.2 (176)	<0.001	DU versus AU	<0.001
					AU versus RR	<0.001
					DU versus RR	0.003
Link worker	32.9 (101)	23.1 (62)	35.6 (151)	0.002	DU versus AU	<0.001
					AU versus RR	<0.001
					DU versus RR	0.446
Attitude towards reception signposting:				<0.001	DU versus AU	<0.001
					AU versus RR	<0.001
					DU versus RR	0.613
Very happy	29.7 (96)	30.3 (81)	21.4 (93)			
Quite happy	41.2 (133)	48.7 (130)	44.9 (195)			
Quite unhappy	18.6 (60)	14.2 (38)	21.4 (93)			
Very unhappy	10.5 (34)	6.7 (18)	12.2 (53)			

ANP = advanced nurse practitioner. MDT = multidisciplinary team. HCA = healthcare assistant. Overall group comparisons used Kruskal–Wallis tests. 2-way comparisons used Mann–Whitney tests.

Percentages differ from n/N due to missing data. Overall percentages (not shown in the table) are given in the main text.

**Table 3.** Awareness of MDT roles comparing patients with versus without multimorbidity; and those aged ≥65 years versus those aged <65 years

	Multimorbid N = 727 % (*n*)	Non-multimorbid N = 322 % (*n*)	*P*-value	Aged ≥65 years N = 530 % (*n*)	Aged <65 years N = 498 % (*n*)	*P*-value
Aware of option of seeing the following MDT roles:						
Nurse (or ANP or HCA)	87.1 (617)	83.8 (263)	0.149	88.6 (467)	83.4 (412)	0.016
Mental health nurse	32.7 (222)	28.6 (88)	0.196	29.2 (145)	33.5 (164)	0.146
Physiotherapist	62.1 (430)	49.0 (149)	<0.001	60.3 (307)	55.8 (271)	0.146
Pharmacist (or technician)	44.7 (310)	36.7 (113)	0.017	43.9 (225)	40.2 (196)	0.226
Link worker	31.0 (213)	32.4 (100)	0.659	27.6 (140)	35.5 (173)	0.007
Attitude towards reception signposting:			0.005			0.439
Very happy	24.5 (173)	30.9 (97)		25.0 (132)	27.8 (137)	
Quite happy	44.7 (316)	44.9 (141)		45.7 (241)	44.0 (217)	
Quite unhappy	19.1 (135)	17.8 (56)		19.0 (100)	18.1 (89)	
Very unhappy	11.7 (83)	6.4 (20)		10.2 (54)	10.1 (50)	

ANP = advanced nurse practitioner. MDT = multidisciplinary team. HCA = healthcare assistant. *P*-values calculated using Mann–Whitney tests.

Percentages differ from n/N due to missing data.

Attitudes towards reception signposting (Table 2) were positive on the whole, with 71% of responders being very (26%) or quite (45%) happy with this system. There was no significant difference between affluent urban and remote and rural areas, but there were significantly more negative views in the deprived urban group (34% very or quite unhappy versus 21% affluent urban versus 29% remote and rural; 3-way comparison P<0.001; pairwise DU vs AU P<0.001, DU vs RR P=0.024). Acceptability of reception signposting (Table 3) was also significantly lower for patients with multimorbidity (31% quite or very unhappy versus 24% in those without; *P*=0.05).

### Interview results

The demographic details and multimorbidity status of the 30 interviewees across the three health boards are given in Supplementary Table S2. The mean age of interviewees was 61.4 years (range 21–83), 22 interviewees had multimorbidity and the mean number of long-term conditions (LTC) was 3.5 (range 0–9). These were similar across health boards.

#### Theme 1: Attitudes towards MDT expansion

The majority (*n* = 22) of 30 patients interviewed were open towards the idea of receiving care from MDT staff for appropriate issues, even though most patients’ direct experience was limited to nurse interactions. Increased availability of appointments and added expertise (in situations such as physiotherapy appointments for musculoskeletal complaints) were perceived benefits of MDT expansion.

*'If* [seeing another professional] *would still get to the root of the problem and potentially negate the long waiting times of waiting for a GP, then, yes, certainly that’s something I’d be interested in.* [For my condition]*, I would be totally happy to see a qualified physio, because that’s going to be better for me than seeing a GP.'* (P10 [SIMD 4, 2 LTCs])

Some patients (*n* = 8) expressed reservations about first-contact MDT care. Such views were expressed in all three population groups (deprived urban *n* = 3; affluent urban *n* = 2; remote and rural *n* = 3), and in patients both with (*n* = 5) and without (*n* = 3) multimorbidity. Many viewed MDT care as a supplement rather than a substitute for holistic, personalised GP care.

*'On one side,* [seeing other professionals] *helps the GP. But on the other side, the GP actually knows you, they see how you are and pick up on not just your ailment but also your mental health.* [The GP] *could be seeing them for, say, a migraine, but there might be a drunken husband or a runaway child.* [If they] *know a wee bit about the history of the family they've got a bit more knowledge. When* [care] *is dotted around different people all seeing you, some of that can get lost, you know? Some of what else is happening in the background can get totally lost. That is just one of the things that worries me* [about these changes]*.'* (P21 [SIMD 4, 9 LTCs])

Access to GP care, especially when patients feel they need it for serious or worrying issues, was an overriding priority for many patients, including those who were more open to MDT care.

*'I still would rather value a GP’s opinion than any of the other healthcare professionals. It does depend very much on what your query is but if you’re feeling really ill I think you need to have a GP examining you. I think you should be listened to carefully about your symptoms before you’re referred to somebody other than the GP.'* (P18 [SIMD 10, 1 LTC])

By contrast, MDT care was felt to be most appropriate for simple, focused, task-oriented interactions.

*'My consultation* [with the nurse practitioner] *was fine because it wasn’t for anything that I felt a doctor should be consulted for. It was to do with a fungal infection on my toenail. So, that’s fine. And I don’t expect to see a doctor for blood tests and things like that …* [But] *when my husband was ill, we saw a nurse practitioner,* [and] *personally I think I would have been happier to see a doctor because of the state of his illness.'* (P22 [SIMD 4, 4 LTCs])

#### Theme 2: Views on reception signposting

While some patients were comfortable being signposted towards MDT care by receptionists, many were unhappy with this system, with 17 out of 30 interviewees expressing concerns. These concerns sometimes related to the nature of the interaction with receptionists (including confidentiality concerns) rather than MDT care itself. Concerns regarding reception signposting were more likely in those patients who were also concerned about GP access, with patients unhappy about feeling challenged by receptionists to justify seeking a GP appointment. Both of these concerns were stronger and more common in deprived urban areas (where they were expressed by a majority) than in remote and rural or affluent urban areas.

*'It’s not their place. They are just the receptionist. I’m not saying they are not intelligent ... but suddenly they have the authority to send you to a physio or refer you to a pharmacist? What authority does a receptionist act on?'* (P26 [SIMD 3, 6 LTCs])

*'I don’t think the onus should be put on the receptionist. It never used to happen. I’m a bit hesitant* [about it]*. I don’t like discussing* [personal issues] *with the receptionist. If you’re making an appointment with the GP, then you obviously feel you need the GP. I don’t make an appointment for the sake of it.* [Reception signposting] *kind of undermines your request.*' (P22 [SIMD 4, 4 LTCs])

On the other hand, many patients (particularly in affluent urban areas) had no objections to the reception signposting system and recognised its value.

*'It’s good if they know who would be the best person to help you. Sometimes, with the training that the receptionists have had, some of them are fantastic. They will say, well, actually it’s probably better if you see this person rather than that person. They are a very valuable member of the team because they know exactly where to point you for you to get the best outcome. So I’m quite happy for them* [to ask]*.'* (P13 [SIMD 8, 5 LTCs])

#### Theme 3: Experiences of MDT care

Almost all patients interviewed had received MDT care and described positive experiences (*n* = 27). Encounters with nurses (either practice nurses or advanced nurse practitioners) were most common (*n* = 20). Physiotherapy (*n* = 10) and pharmacy (*n* = 7) consultations were also familiar to many patients, and were felt to be particularly beneficial in terms of perceived expertise and reduced time pressure.

*'The physio was very attentive, told you what to do and why you had to do it. They explained things a bit better* [than the GP]*. That’s what I felt anyway. It was much easier* [to arrange that appointment]. *Face-to-face, straight away, done and dusted. And it’s an easier experience seeing another healthcare professional, it’s a more relaxed experience. I'm not saying the GP is a bad experience, but it’s often, “what’s wrong with you? Aye, this, that, that’s fine, oh aye”. That’s it and away you go, you know what I mean?* [With the physiotherapist] *there was more time.'* (P26 [SIMD 3, 6 LTCs])

Mental health nurse (*n* = 2) and link worker (*n* = 1) interactions were least common, occurring only in practices serving deprived urban areas, but the value of these was also evident.

*'When I started to go on this Universal Credit, because I didn’t understand it, there was someone in the practice* [a Link Worker] t*hat explained it to me and told me what I was entitled to and helped me get what I was entitled to … unbelievable … seriously, really helpful.' (*P6 [SIMD 1, 2 LTCs])

### Synthesis of quantitative and qualitative results

Table 4 gives the convergence coding matrix derived from the triangulation of survey and interview results. There was a high degree of convergence on several findings. Some themes covered in the interviews did not have corresponding data in the survey (coded as ‘silence’ on the matrix) because interview occurred after the survey and explored wider, contextualising questions. There was also some divergence of findings, specifically that the high awareness of pharmacy and physiotherapy roles reported by survey responders in remote and rural areas was not reflected in the interviews. This is likely owing to the small sample size of interviewees.

**Table 4.** Triangulation matrix for the findings from the quantitative and qualitative components of the study

Themes	Survey findings	Interview findings	Convergence coding
Awareness of the availability of MDT appointments	High awareness of nurse appointments (86%). Intermediate awareness of pharmacy (42%) and physiotherapy (58%) roles. Low awareness of MHN (32%) and LW (31%) appointments.	Most patients had experience of nurse appointments (*n* = 20). Physiotherapy (*n* = 10) and pharmacy (*n* = 7) were familiar to many. MHN (*n* = 3) and LW (*n* = 1) interactions were less common.	Convergence
Differences in MDT-role awareness between patient groups	High awareness of nurse appointments in all groups, but significantly higher in RR areas. Awareness of physiotherapy and pharmacy roles was highest in RR areas. Awareness of MHN and LW roles was lowest in AU areas, and similar in DU and RR areas. Awareness of physiotherapy and pharmacy roles higher in patients with multimorbidity than without.	Experience of nurse interactions were common in patients across all population groups (DU, AU, and RR). Most physiotherapy interactions were reported by patients in DU areas. Most pharmacy interactions were reported by patients in AU areas. MHN and LW interactions were only reported by patients in DU areas. Nine interviewees (out of 30) were non-multimorbid, of which only one had experience of pharmacy or physiotherapy care. Twenty-one interviewees were multimorbid, of which 16 had experience of pharmacy or physiotherapy care.	Partial convergence Divergence *Contrary to the survey, interviewees from RR areas had comparatively little experience of physiotherapy or pharmacy care*. Partial convergence Convergence
Acceptance of reception signposting	Attitudes towards reception signposting were favourable overall (71% quite or very happy). This was significantly lower in patients in DU areas.	Most patients (*n* = 20) were, on balance, supportive of reception signposting, but many (*n* = 16) raised concerns. These concerns were more common in DU areas, than RR or AU.	Convergence
Attitudes towards MDT care	No data	Most interviewees were open to the role of MDT in primary care. Some expressed reservations about MDT appointments as an alternative to GP care, citing the importance of GP access and holistic, relationship-based care.	Silence *Attitudes towards MDT care were only explored in qualitative interviews, so there is silence on this in the quantitative component of the study*.
Experiences of MDT care	No data	Experiences of MDT care were overwhelmingly positive and covered all types of MDT staff.	Silence *Experiences of MDT care were only explored in qualitative interviews, so there is silence on this in the quantitative component of the study*.

AU = affluent urban. DU = deprived urban. LW = link worker. MDT = multidisciplinary team. MHN = mental health nurse. RR = remote and rural.

These five themes arising from the synthesis of survey and interview findings differ from the three broader themes identified in the preceding qualitative analysis of interviews.

## Discussion

### Summary

Four years since the introduction of the new GP contract in Scotland, MDT care has expanded with limited patient awareness, but patients do recognise its potential value. Patients who had received MDT care reported positive experiences and highlighted improved access and added expertise as perceived benefits. However, many patients were unhappy with reception signposting to first-contact MDT care, especially those in deprived urban areas living with multimorbidity. Access to GP care, especially when patients feel they need it for serious or worrying issues, was an overriding priority for many patients, and MDT care was seen as a supplement rather than a substitute for this. Concerns about reception signposting were more likely in those patients who were also concerned about GP access.

There were also differences in patients’ awareness of MDT roles across geographic and socioeconomic groups, as well as by age and multimorbidity. Pharmacy and physiotherapy roles were most familiar to patients with multimorbidity and those in remote and rural areas, while awareness of mental health nurses and link workers was lowest in affluent urban areas. Patients aged ≥65 years had significantly lower awareness of link workers (P = 0.007).

### Strengths and limitations

The strengths of this study include its use of mixed methodology to explore and evaluate patient views, the relatively large survey sample, and the inclusion of three populations of interest. The strength of the survey findings are limited by the low response rate of 17% (12% deprived urban versus 27% affluent urban versus 20% remote and rural), although this is not dissimilar to that seen in Scottish Government patient surveys.^31^ Of note, the deprived urban group, which, like in other surveys, had a much lower response rate, was the biggest group in our sample, comprising 58% of distributed surveys. The response rates may also have been limited by the use of a postal survey, rather than in-person data collection at the GP practice, a method that was dictated by the pandemic. Additionally, the funding constraints of the study meant that postal reminders were not possible.

Survey responders differed from non-responders in terms of age in all groups (deprived urban, affluent urban, and remote and rural). In the deprived urban group, responders were also significantly less deprived than non-responders (Supplementary Table S1). While this affects the generalisability of the survey findings, it may in fact mean that the differences found between the deprived urban and affluent urban groups are an underestimate, as those responding were the 'least deprived of the deprived'.

With respect to the qualitative methods, the reliance on telephone rather than face-to-face interviews as a result of the pandemic may have affected the quality of evidence obtained, given the absence of non-verbal communication, although research suggests there is little difference in quality between these modes of interview.^32^ The recruitment of interviewees from a volunteer subset of questionnaire responders creates a risk of bias since this method may give prominence to those with strong views. However, while our findings do not claim to be fully generalisable, the number of interviews conducted, the purposive sampling method, and the saturation of data suggest that our findings give a valuable representation of the range of views held by patients across geographic and socioeconomic groups and, in particular, older patients with multimorbidity.

The study would be strengthened by the inclusion of data on practice-level differences in MDT care availability, reception signposting methods, and appointment booking systems, as these differences are likely to have influenced patients’ responses. Local variations in the implementation of the contract have been reported, including the provision of MDT services in some areas through ‘hubs’ rather than individual GP practices.^19,33^ However, it was not possible to collect data on these practice-level differences within the resource constraints of the study. The high level of awareness of nurse appointments is also likely to have been skewed by the broad definition of ‘nurses’ used in the survey (which covered practice nurses, advanced nurse practitioners, and the support role of healthcare assistants, who are not registered nurses), limiting the interpretation of this finding.

### Comparison with existing literature

The differences found in MDT awareness between deprived urban, affluent urban, and remote and rural areas likely reflects differing exposure to MDT care owing to differences in individuals’ health needs and in practices’ MDT provision. Lower MDT awareness in affluent urban areas, for example, is in keeping with the lower frequency of GP attendance, better general health, and lower levels of multimorbidity in these areas, than deprived urban and remote and rural areas.^21,25,34,35^ However, significantly higher awareness of mental health nurses was found in remote and rural than affluent urban areas, despite the fact that mental–physical multimorbidity and levels of anxiety and depression are similar between affluent urban and remote and rural areas.^21^ The reasons for this discrepancy are not clear.

A range of challenges affecting the expansion and integration of primary care MDTs in Scotland was highlighted in our recent qualitative evaluations of the views of primary care professionals and stakeholders.^19,20^ The importance of involving and engaging patients in the redesign of primary care services has been highlighted by the Organisation for Economic Cooperation and Development (OECD), but our recent systematic scoping review of OECD countries and China found limited evidence of it happening.^^13,36^^

Both the levels of awareness of MDT roles and the acceptability of reception signposting found in this study are very similar to those found in a recent Scottish Government survey with a nationally representative sample of >1000 patients.^37^ This also showed that patients view the GP as the first point of contact and prefer signposting by a GP, results that echo the findings of our qualitative interviews. Additionally, the Scottish Government survey found high levels of trust in the advice of nurses, physiotherapists, and pharmacists in primary care (76%, 66%, and 65%, respectively, compared with 75% with complete trust in GPs and 52% in mental health nurses).^37^ These results fit with our interview findings of positive experiences of care for these three MDT roles in particular.

A number of international reviews have highlighted the paucity of evidence evaluating patients’ views and experiences of MDT care.^8,13,22^ However, our findings of broadly positive patient experiences are consistent with a recent integrative review of 48 international studies.^23^ Interestingly, and in close alignment with our study, as well as finding generally positive patient experiences of MDT care, this review highlighted concerns that increased access to MDT care did not necessarily increase care quality, and that MDT care was *'inconsistently holistic*'.^23^

Evidence on patients’ views regarding reception signposting is also lacking, although a recent study of staff views reported concerns over the lack of clarity over receptionists’ role and remit, and the need for appropriate training and development.^38^ These findings echo the reservations expressed by patients in our study. While this paper focuses on the views of patients with respect to MDT expansion, we have also published a more comprehensive summary of patients’ views and experiences of primary care in Scotland.

### Implications for research and practice

MDT expansion is a core feature of not only the Scottish GP contract, but also of primary care reforms in healthcare systems elsewhere globally,^13^ and the views and experiences of patients with respect to this are under-explored.^8,13,22^ Consequently, this study provides an important insight for all primary care systems undergoing similar changes.

The implementation of the new GP contract in Scotland has clearly been affected by the pandemic, and other challenges to MDT expansion have been identified,^19,20^ but further work is needed to assess variations in the availability of primary care MDT services across Scotland, as this has implications for interpreting differences in patient views on these reforms. Our findings, however, suggest that, despite limited awareness of MDT expansion, it has qualified acceptance and generally leads to positive patient experiences.

However, access to GP care remains an overriding priority for patients and MDT care is seen as a supplement rather than a substitute for this. Concerns about reception signposting to first-contact MDT care are especially strong in patients from deprived areas with multimorbidity. These findings represent a barrier to the expansion of first-contact MDT care, and therefore to the new GP contract’s aim of reducing GP workload and freeing-up time for GPs to spend with the most complex patients. Recent research, including our qualitative studies with professionals, suggest this is not yet happening.^19,20,39^ Given that this is a key mechanism by which the contract seeks to reduce health inequalities, these issues must be addressed if continued MDT expansion is to benefit those with the most complex needs.
